# Deep Herpes in an Adolescent With Severe Atopic Dermatitis and Concurrent Methicillin-Resistant Staphylococcus aureus (MRSA) Bacteremia

**DOI:** 10.7759/cureus.115731

**Published:** 2026-09-03

**Authors:** Johanna Graciella M Nalupta, Neha Bhagi, Vesna Petronic-Rosic

**Affiliations:** 1 Pediatrics, John H. Stroger, Jr. Hospital of Cook County, Chicago, USA; 2 Dermatology, John H. Stroger, Jr. Hospital of Cook County, Chicago, USA

**Keywords:** adolescent, deep herpes, dermatopathology, herpes simplex virus, mrsa bacteremia

## Abstract

Herpes simplex virus (HSV) infection is a well-recognized complication of atopic dermatitis, most commonly presenting as eczema herpeticum. Deep herpes is a recently described clinicopathologic manifestation of HSV infection, characterized by viral involvement of cutaneous stromal cells with limited or absent epidermal infection, which can make diagnosis challenging when classic histopathologic findings are absent.

We report a 17-year-old adolescent with severe atopic dermatitis who presented with fever and a rapidly progressive painful erosive eruption. Evaluation revealed methicillin-resistant *Staphylococcus aureus* (MRSA) bacteremia. Skin biopsy demonstrated a hyperplastic spongiotic epidermis with a superficial and mid-dermal inflammatory infiltrate. Although characteristic epidermotropic HSV cytopathic changes were absent, HSV-2 immunohistochemistry identified infected cells within the dermal inflammatory infiltrate, supporting a diagnosis of deep herpes. The patient demonstrated rapid clinical improvement following treatment with intravenous acyclovir and vancomycin, with cessation of new lesion formation, progressive re-epithelialization, and resolution of systemic symptoms.

This case highlights deep herpes as an uncommon manifestation of HSV infection in an adolescent with severe atopic dermatitis complicated by concurrent MRSA bacteremia. Pediatricians should consider deep herpes in patients with severe atopic dermatitis who present with painful erosive eruptions and systemic symptoms, particularly when clinical findings and routine diagnostic testing are inconclusive.

## Introduction

Deep herpes is a recently recognized clinicopathologic pattern of herpesvirus infection characterized by viral involvement of dermal and subcutaneous stromal cells with limited or absent epidermal infection [1]. In the largest series to date, Krystel-Whittemore and colleagues demonstrated that affected patients often lack the classic epidermotropic viral cytopathic changes traditionally associated with herpes simplex virus (HSV) infection, making diagnosis particularly challenging [1]. Consequently, recognition frequently depends on close clinicopathologic correlation and immunohistochemical confirmation [1]. In contrast, conventional eczema herpeticum is characterized predominantly by epidermotropic HSV infection with viral cytopathic changes involving epidermal keratinocytes [2,3].

Patients with atopic dermatitis are at increased risk of viral and bacterial infections due to epidermal barrier dysfunction and immune dysregulation [2,3]. However, many patients described with deep herpes have had additional immunocompromising conditions, including malignancy, transplantation, autoimmune disease, or immunosuppressive therapy. The full clinical spectrum and risk factors for deep herpes remain incompletely defined, and reports in pediatric patients are especially limited [1].

We report an adolescent with severe atopic dermatitis who developed HSV-2-associated deep herpes complicated by concurrent methicillin-resistant *Staphylococcus aureus* (MRSA) bacteremia. The pediatric presentation, identification of HSV-2 rather than HSV-1, absence of traditional systemic immunosuppression, and concurrent invasive bacterial infection represent distinctive features of this case and expand the limited reported clinical spectrum of deep herpes. 

## Case presentation

A 17-year-old male with a history of moderate-to-severe atopic dermatitis initially presented to an outside hospital with a seven-day history of a rapidly progressive painful erosive eruption associated with fever (maximum temperature 101.3°F) and malaise. The eruption initially developed on the buttocks in areas of active dermatitis before spreading over several days to involve the trunk and extremities. He was subsequently transferred to our institution for further evaluation and management. The patient denied oral ulcerations, ocular symptoms, recent medication exposure, or known sick contacts. There was no history of primary immunodeficiency, malignancy, organ transplantation, or systemic immunosuppressive therapy. 

His dermatologic history was notable for chronic, poorly controlled atopic dermatitis characterized by recurrent flares requiring intermittent topical corticosteroid therapy. He had experienced previous episodes of secondary bacterial skin infection but had no documented history of herpes simplex virus infection.

On presentation, the patient appeared ill but remained hemodynamically stable. Cutaneous examination demonstrated numerous erosive and crusted lesions involving the upper and lower extremities as well as the trunk. Many lesions exhibited hemorrhagic crusting and were associated with significant tenderness. Background findings included diffuse xerosis, lichenification, and chronic eczematous changes consistent with longstanding atopic dermatitis. No intact vesicles or bullae were identified. Mucosal surfaces, including the oral cavity, were spared. Nikolsky sign was negative (Figure 1). 

**Figure 1:**
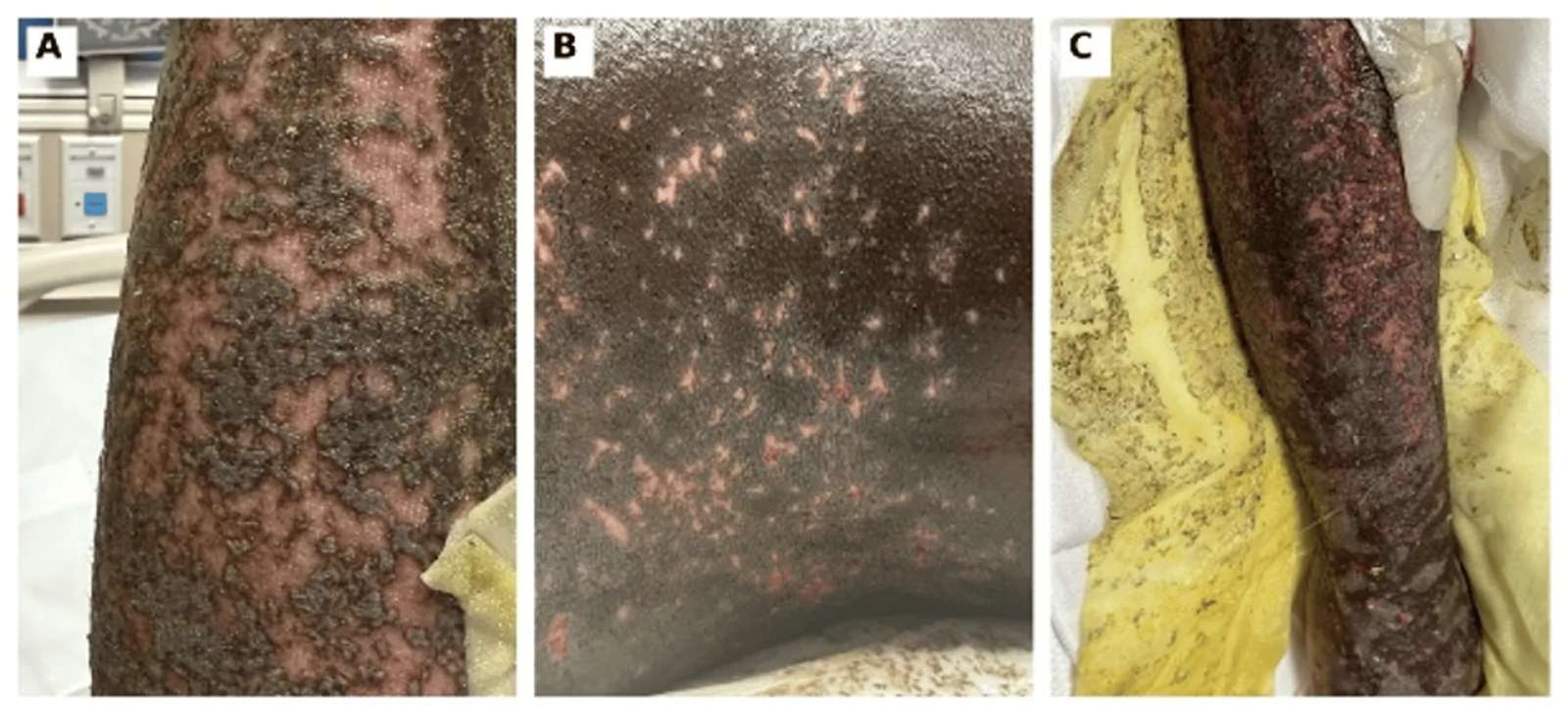
Clinical presentation. (A)-(C) Widespread punched-out erosions and hemorrhagic crusted plaques involving the extremities and trunk in an adolescent with severe atopic dermatitis.

Given the extensive skin involvement, fever, severe pain, and atypical morphology, initial clinical considerations included eczema herpeticum, bullous impetigo, and severe bacterial superinfection of atopic dermatitis. The absence of intact vesicles, lack of mucosal involvement, and indeterminate HSV polymerase chain reaction (PCR) testing complicated the diagnostic evaluation and prompted histopathologic examination. The patient was admitted for further evaluation and management. 

Laboratory evaluation demonstrated elevated inflammatory markers, including C-reactive protein (10.06 mg/dL; reference range, 0-0.5 mg/dL) and procalcitonin (12.04 ng/mL; laboratory reference interpretation: <0.5 ng/mL, low risk of severe sepsis; >2.5 ng/mL, high risk of severe sepsis), as well as hypoalbuminemia (2.9 g/dL; reference range, 3.8-5.2 g/dL). The white blood cell count was within normal limits, with differential analysis demonstrating relative monocytosis (16.9%) and eosinophilia (8.3%).

Cultures obtained from cutaneous lesions grew methicillin-sensitive *Staphylococcus aureus* and *Pseudomonas aeruginosa*, whereas one admission blood culture set was positive for methicillin-resistant *Staphylococcus aureus* (MRSA). MRSA was additionally detected by nucleic acid amplification testing. Susceptibility testing demonstrated resistance to cefazolin and oxacillin and susceptibility to vancomycin and daptomycin.

Lesional HSV PCR testing obtained at the referring outside hospital prior to transfer and before initiation of acyclovir therapy was reported as indeterminate. Additional details regarding the sampling method, specimen adequacy, assay parameters, and the laboratory-specific reason for classification as indeterminate were not available in the transferred medical records. The result therefore neither established nor excluded HSV infection, prompting further tissue-based evaluation after transfer.

Histopathologic findings 

Histopathologic examination of the punch biopsy demonstrated hemorrhagic scale crust containing neutrophils and a small number of Gram-positive bacteria overlying a hyperplastic and spongiotic epidermis. A moderately dense superficial and mid-dermal perivascular inflammatory infiltrate was present. 

Approximately twelve cells demonstrated positive immunohistochemical staining for HSV-2. These HSV-2-immunoreactive cells morphologically resembled lymphocytes and were distributed interstitially within the reticular dermis. HSV-2 immunoreactivity was not identified within epidermal keratinocytes. Immunohistochemical staining for HSV-1 was negative, and Epstein-Barr virus in situ hybridization was likewise negative (Figure 2). 

**Figure 2:**
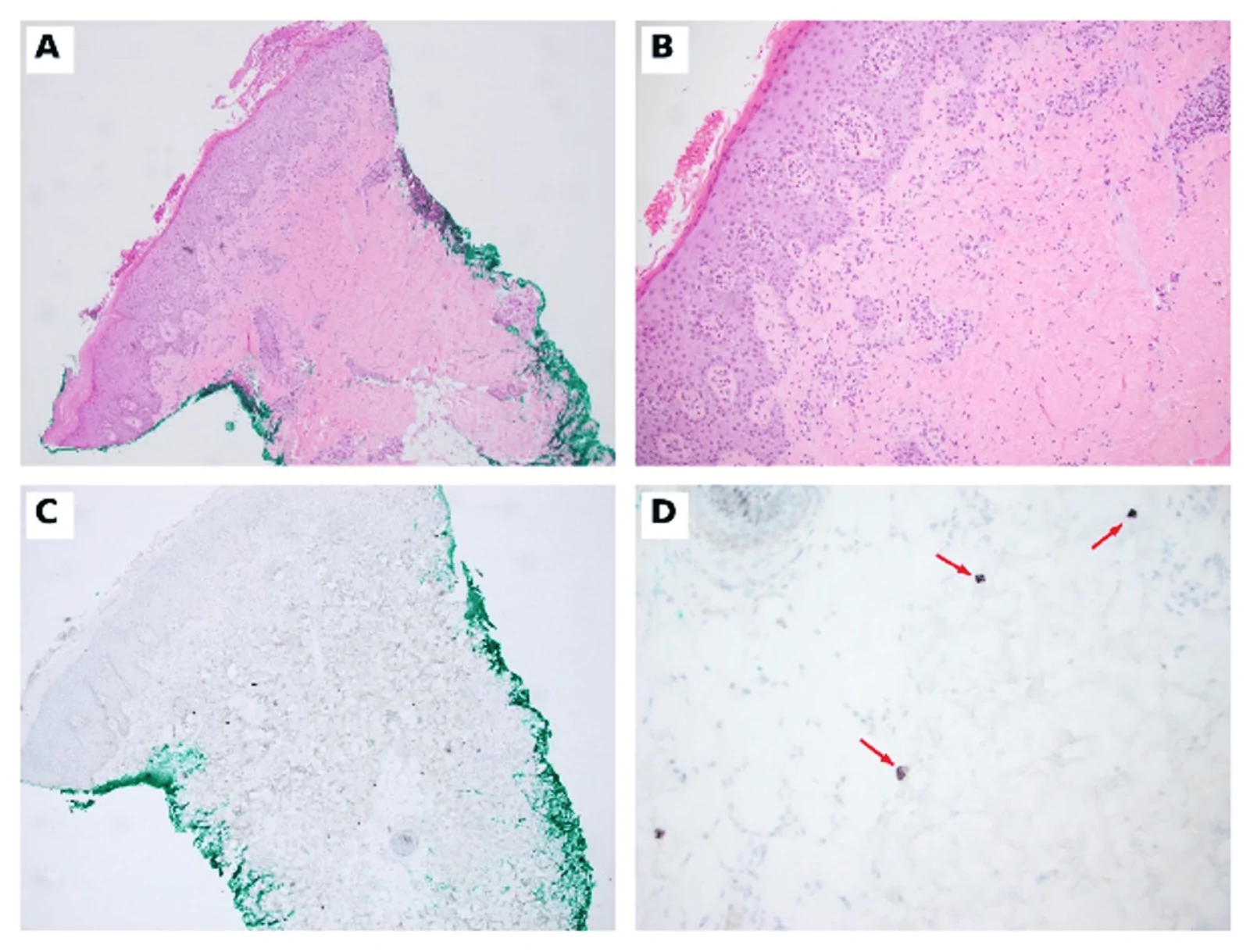
Histopathologic findings. (A) H&E, 2×, and (B) H&E, 10×: Punch biopsy of the right dorsal forearm demonstrating a hyperplastic and spongiotic epidermis with a superficial and mid-dermal moderately dense perivascular inflammatory infiltrate. (C) HSV-2 immunohistochemistry, 2×, and (D) HSV-2 immunohistochemistry, 20×: HSV-2 immunohistochemistry highlights scattered immunoreactive cells within the dermis (red arrows), supporting the diagnosis of deep herpes. HSV-1 immunohistochemistry and Epstein-Barr virus in situ hybridization were negative.

Notably, diagnostic epidermotropic HSV cytopathic changes were absent. Although the inflammatory infiltrate was moderate rather than dense, HSV-2 immunohistochemical staining was unequivocal. Approximately twelve HSV-2-immunoreactive cells, morphologically resembling lymphocytes, were identified interstitially within the reticular dermis, while HSV-2 immunoreactivity was absent from epidermal keratinocytes. Based on these findings, the dermatopathologist interpreted the biopsy as suggestive of deep herpes and recommended clinicopathologic correlation. The absence of classic epidermotropic viral cytopathic changes, coupled with HSV-2 immunoreactivity within dermal inflammatory cells, supported the diagnosis of deep herpes [1].

Because no standardized diagnostic protocol for deep herpes has been established, the diagnosis in this case was based on clinicopathologic correlation. The diagnostic evaluation included lesional HSV PCR, punch biopsy with routine histopathologic examination, and ancillary viral studies, including HSV-1 and HSV-2 immunohistochemistry and Epstein-Barr virus in situ hybridization. Taken together with the clinical presentation, these findings supported the diagnosis of deep herpes.

Clinical course and management 

The patient therefore had concomitant HSV-2 infection and MRSA bacteremia requiring concurrent antiviral and antibacterial therapy. Given concern for disseminated herpesvirus infection in the setting of extensive painful erosions and fever, empiric intravenous acyclovir 500 mg every eight hours was initiated on hospital day one. Intravenous acyclovir was continued for seven days, after which the patient was transitioned to oral valacyclovir 1 g every 12 hours to complete a total 14-day course of antiviral therapy.

Concurrent MRSA bacteremia was treated with intravenous vancomycin 1 g every 12 hours beginning on hospital day one. Two repeat blood cultures obtained on hospital days three and four were negative, documenting clearance of bacteremia. Intravenous vancomycin was continued through hospital day 11, after which the patient was transitioned to oral trimethoprim-sulfamethoxazole (Bactrim DS), two tablets twice daily, to complete approximately 14 days of antibacterial therapy following documented clearance of bacteremia. No additional evaluation for metastatic or endovascular complications was pursued because bacteremia cleared on serial blood cultures and the patient demonstrated continued clinical improvement without signs or symptoms suggestive of a metastatic focus of infection.

Renal function was monitored during concomitant acyclovir and vancomycin therapy and remained stable. At baseline, blood urea nitrogen (BUN) was 11 mg/dl and serum creatinine was 0.9 mg/dl. On hospital day three, BUN was 9 mg/dl and serum creatinine remained 0.9 mg/dl. Near completion of intravenous therapy, BUN was 16 mg/dl and serum creatinine remained 0.9 mg/dl. Overall, BUN and serum creatinine remained stable throughout treatment, with no evidence of treatment-associated acute kidney injury. 

The patient underwent multidisciplinary evaluation by dermatology and infectious disease specialists. By approximately one week after initiation of antiviral and antibacterial therapy, the patient demonstrated progressive clinical improvement (Figure 3). No new erosive lesions developed, and existing lesions gradually re-epithelialized with residual post-inflammatory hypopigmentation. Systemic symptoms resolved completely, and continued follow-up was arranged to optimize long-term management of atopic dermatitis.

**Figure 3:**
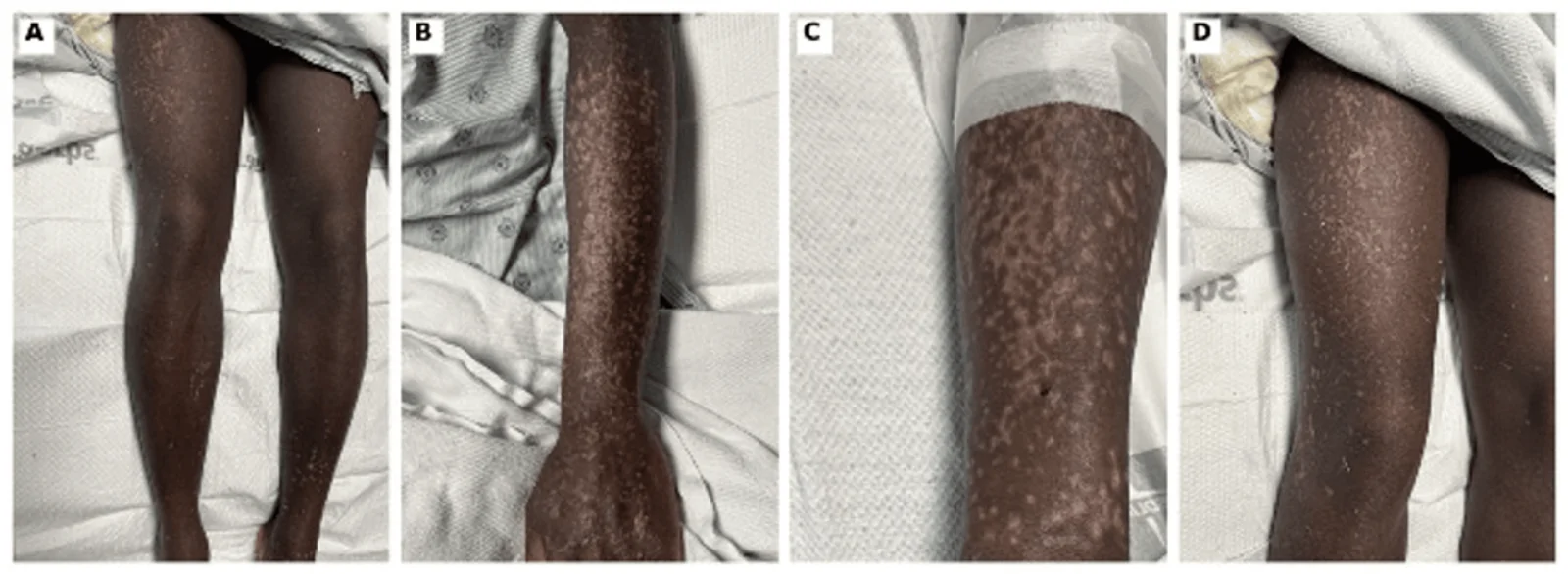
Clinical improvement following treatment. (A)-(D) Interval resolution of erosive lesions with re-epithelialization and residual post-inflammatory hypopigmentation involving the upper and lower extremities following initiation of antiviral and antibiotic therapy. The patient was discharged after continued clinical improvement with plans to complete antiviral and antibacterial therapy and close outpatient follow-up.

## Discussion

Deep herpes is a recently recognized clinicopathologic manifestation of herpes simplex virus (HSV) and varicella-zoster virus (VZV) infection characterized by viral involvement of cutaneous stromal cells with limited or absent epidermal keratinocyte infection [1]. In their landmark multicenter study, Krystel-Whittemore and colleagues described herpesvirus infection involving stromal cells of the dermis and subcutis, expanding the traditional understanding of herpesvirus infection as a predominantly epidermotropic process [1]. Importantly, many cases lacked the classic epidermal viral cytopathic changes typically associated with HSV infection, creating potential diagnostic challenges for both clinicians and pathologists [1]. 

The clinicopathologic features of our case closely mirror those reported in the original deep herpes series and support classification of this eruption as deep herpes rather than conventional epidermotropic HSV infection. Approximately twelve HSV-2-immunoreactive cells, morphologically resembling lymphocytes, were distributed interstitially within the reticular dermis, while HSV-2 immunoreactivity was absent from epidermal keratinocytes. This pattern, together with the absence of diagnostic epidermotropic viral cytopathic changes, supported the diagnosis of deep herpes [1]. Because no standardized diagnostic protocol for deep herpes has been established, clinicopathologic correlation and immunohistochemistry were particularly informative when clinical findings and lesional PCR were inconclusive [1,4].

The evidence supporting deep herpes as a distinct clinicopathologic entity remains limited, with much of the current understanding derived from a single multicenter series [1]. Its incidence, risk factors, pathogenesis, and full clinical spectrum therefore remain incompletely defined, emphasizing the need for additional validation.

The pathogenesis of deep herpes remains incompletely understood. Stromal herpes disease has long been recognized in ophthalmology [5], and experimental studies demonstrate that HSV-1 can infect stromal cells [6]. Viral interactions with mesenchymal stromal cells may also influence local immune responses [7]. These observations provide biological plausibility for cutaneous stromal HSV infection, although the mechanism responsible for this distribution in deep herpes remains unknown.

Severe atopic dermatitis may have contributed to susceptibility in this patient. Atopic dermatitis is associated with epidermal barrier dysfunction and immune dysregulation [3]. Loss-of-function variants affecting filaggrin contribute to impaired epidermal integrity [8], while reduced antimicrobial peptide expression may further increase susceptibility to cutaneous infection [9]. Patients with atopic dermatitis are susceptible to bacterial and viral infections, including *S. aureus *and HSV [2]. However, filaggrin mutation status and serum IgE were not assessed in this patient; therefore, their specific contribution remains theoretical rather than directly demonstrated. Clinically, the severity of the patient's atopic dermatitis supported substantial epidermal barrier dysfunction as a potential predisposing factor. 

Concurrent MRSA bacteremia added diagnostic and therapeutic complexity. Bacterial infection may have contributed to the inflammatory findings on histopathology and potentially obscured subtle viral changes. Nevertheless, discrete HSV-2-immunoreactive cells within the reticular dermis supported concomitant HSV-2 infection. Both infections required targeted treatment, with antiviral and antibacterial therapies administered concurrently. Consequently, the patient's subsequent clinical improvement cannot be attributed to either therapy alone or used independently to confirm the diagnosis of deep herpes.

The indeterminate lesional HSV PCR despite positive HSV-2 immunohistochemistry is particularly instructive. The specimen was obtained at the referring outside hospital before initiation of antiviral therapy; however, details regarding the sampling technique, specimen adequacy, assay parameters, and the laboratory-specific reason for classification as indeterminate were unavailable. Conventional cutaneous HSV infection characteristically demonstrates epidermal viral cytopathic changes [4], whereas deep herpes may involve deeper dermal cells with limited or absent epidermal infection [1]. One possible explanation is that deeper localization of infected cells could reduce the amount of viral material available to superficial lesional sampling. However, this remains speculative, and technical or sampling factors cannot be excluded. A single case cannot establish an association between dermal localization and reduced HSV PCR sensitivity.

Deep herpes may mimic eczema herpeticum, bullous impetigo, and bacterial superinfection of atopic dermatitis (Table 1). The absence of intact vesicles, indeterminate HSV PCR, and concurrent bacterial infection made clinical recognition particularly difficult in this patient. This case highlights the potential value of clinicopathologic correlation and tissue-based testing when clinical findings and routine virologic testing are inconclusive [1,4].

**Table 1:** Differential diagnosis of painful erosive eruptions in severe atopic dermatitis. HSV: herpes simplex virus; PCR: polymerase chain reaction.

Feature	Deep herpes	Eczema herpeticum	Bullous impetigo	Bacterial superinfection of atopic dermatitis
Pain	Prominent	Common	Mild to moderate	Variable
Intact vesicles	Often absent	Common	Bullae present	Usually absent
Fever/systemic symptoms	May occur	Common	Less common	Variable
HSV PCR	Performance not well established; may be inconclusive	Commonly used for diagnostic confirmation	Negative	Negative
Histopathology	HSV-positive dermal inflammatory/stromal cells with absent epidermotropic changes	Epidermotropic HSV cytopathic changes	Subcorneal neutrophilic blister with bacteria	Acute bacterial inflammation
HSV immunohistochemistry	May provide diagnostic support	Supportive	Negative	Negative
Key diagnostic pitfall	Lack of classic epidermal HSV changes	May mimic bacterial infection	May mimic HSV infection	May obscure concomitant HSV infection

Reports of deep herpes in pediatric patients remain limited [1]. This case expands the reported clinical spectrum by describing HSV-2-associated disease in an adolescent with severe atopic dermatitis and concurrent MRSA bacteremia. However, these observations are based on a single case and should be considered hypothesis-generating rather than evidence of a causal relationship among atopic dermatitis, HSV-2 infection, and deep dermal viral localization. Further cases are needed to better define the clinical and diagnostic significance of this recently described pattern.

## Conclusions

Deep herpes is a recently recognized pattern of herpesvirus infection that may be difficult to diagnose when classic clinical and histopathologic features of HSV infection are absent. This case describes HSV-2-associated deep herpes in an adolescent with severe atopic dermatitis and concurrent MRSA bacteremia, with HSV-2 immunoreactivity identified within reticular dermal lymphocytes despite an indeterminate lesional HSV PCR.

This case highlights the potential value of clinicopathologic correlation and immunohistochemistry when routine testing is inconclusive. In this patient, HSV-2 immunohistochemistry provided important diagnostic information despite an indeterminate lesional HSV PCR. However, findings from a single case cannot establish the sensitivity, specificity, or broader diagnostic significance of HSV-2 immunohistochemistry in deep herpes. The proposed relationship between deep dermal viral localization and inconclusive HSV PCR testing remains hypothesis-generating and requires validation in larger studies.

## References

[1] Krystel-Whittemore M, Chan MP, Shalin SC (2021). Deep herpes. Am J Surg Pathol.

[2] Leung DY, Bieber T (2003). Atopic dermatitis. Lancet.

[3] Ong PY, Leung DY (2016). Bacterial and viral infections in atopic dermatitis: a comprehensive review. Clin Rev Allergy Immunol.

[4] Hoyt B, Bhawan J (2014). Histological spectrum of cutaneous herpes infections. Am J Dermatopathol.

[5] McGill J (1987). The enigma of herpes stromal disease. Br J Ophthalmol.

[6] Baldwin J, Park PJ, Zanotti B (2013). Susceptibility of human iris stromal cells to herpes simplex virus 1 entry. J Virol.

[7] Thanunchai M, Hongeng S, Thitithanyanont A (2015). Mesenchymal stromal cells and viral infection. Stem Cells Int.

[8] Palmer CN, Irvine AD, Terron-Kwiatkowski A (2006). Common loss-of-function variants of the epidermal barrier protein filaggrin are a major predisposing factor for atopic dermatitis. Nat Genet.

[9] Ong PY, Ohtake T, Brandt C (2002). Endogenous antimicrobial peptides and skin infections in atopic dermatitis. N Engl J Med.

